# Lumbar Fetiform Teratoma: Limb in the Back – A case report

**DOI:** 10.1016/j.radcr.2021.11.037

**Published:** 2021-12-15

**Authors:** Mohammad Tahir Aien, Naqibullah Foladi, Mohammad Saboor Rastin, Wajihullah Walizada

**Affiliations:** a Radiology Department, Kabul University of Medical sciences, Kabul, Afghanistan; b Radiology Department, French Medical Institute for Mothers and Children (FMIC), behind Kabul University of Medical Sciences (KUMS), Kabul, Afghanistan; c Radiology Department, French Medical Institute for Mothers and Children (FMIC), Kabul, Afghanistan; d Pediatrics Department, French Medical Institute for Mothers and Children (FMIC), Kabul, Afghanistan

**Keywords:** Fetiform teratoma, Fetus in fetu, Case report

## Abstract

Teratoma is a congenital neoplasm deriving from one or more embryonic layers. Fetiform teratoma is a highly differentiated rare type of teratoma. The authors present a 1-day-old neonate for a lumbar region mass, grossly appearing like an extremity. CT scan was performed showing a highly organized extremity skeleton in the lumbar region consisting of flat, long and short bones. Fetiform teratoma should be differentiated from a fetus in fetu, as the former lacks axial skeleton while it is the main feature of the latter, respectively. Both have different prognostic implications, and surgical excision is the treatment of choice.

## Introduction

Teratoma is derived from the Greek word teras meaning monster and onkoma meaning swelling. Teratomas contain various types of differentiated tissues such as hair, skin, teeth. Few entities have to be put into consideration when dealing with fetal tumors, such as mature teratoma, fetiform teratoma, and fetus in fetu [1]. Fetiform teratoma is a rare subtype of mature teratoma consisting of highly differentiated tissues and limbs [2,3] lacking axial skeleton, visceral organs, and skeletal muscles [1,4]. Authors present a case of neonate presenting with a lumbar mass resembling an extremity demonstrating highly organized flat bones, long bones, and digits.

## Case presentation

**Fig. 1:**
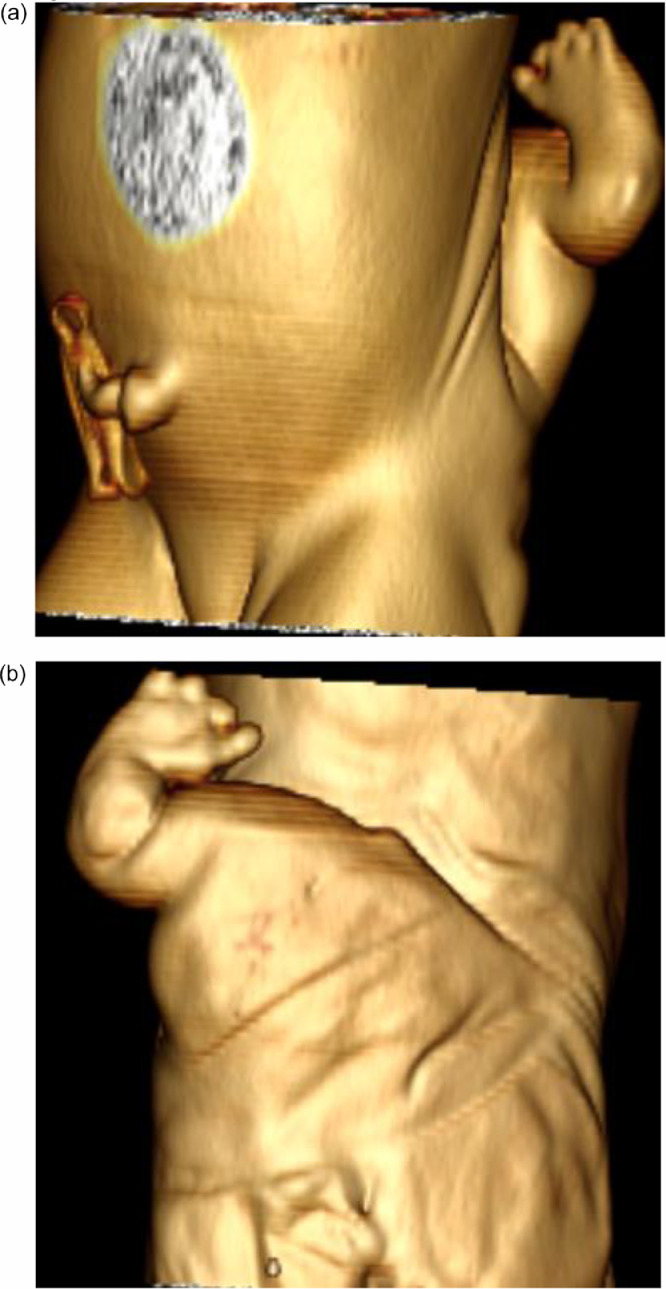
([1]and [2]) of 3D VRT images demonstrate lumbar mass resembling a limb

**Fig. 2:**
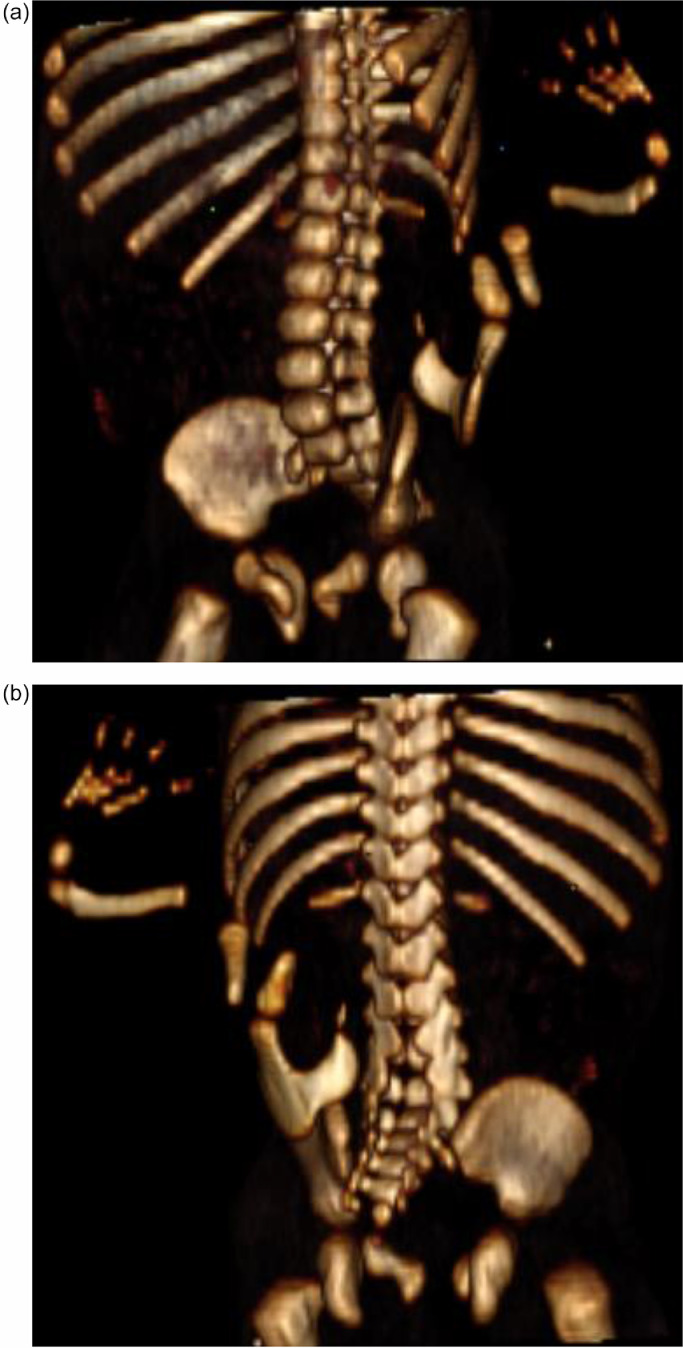
(2A and 2B) of 3D VRT images demonstrate highly developed skeleton, consisting of flat bones, long bone and digits near the lumbar spine

**Fig. 3:**
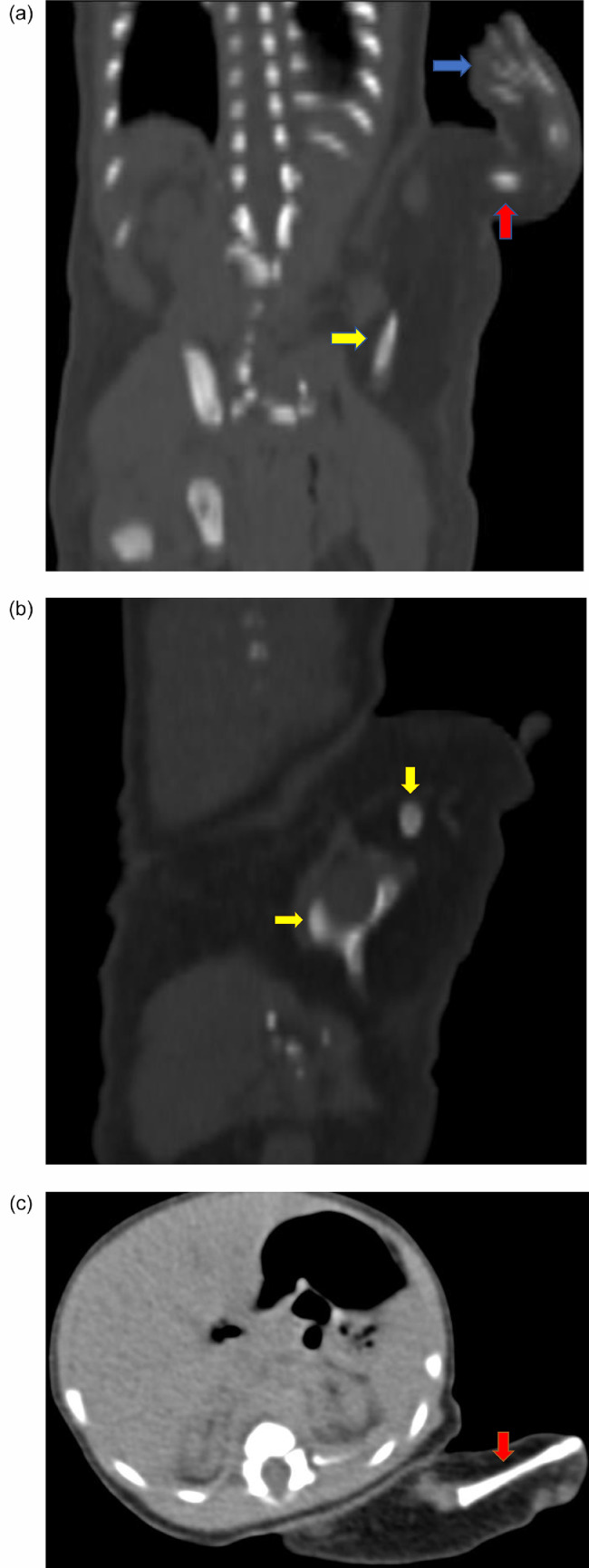
(3A – 3C) selected coronal, sagittal and axial bone and soft tissue window images demonstrate limb with digits (blue arrow), flat bones (yellow arrows), and long bone (red arrows)

A 1-day-old female neonate patient with lumbar mass was referred to the radiology department for further workup of the lesion. Physical examination revealed an extremity-like mass in the lower back. There was no pertinent prior medical, family, and psycho-social history including any genetic predisposition. The abdomen CT scan demonstrated a highly developed skeleton consisting of highly differentiated flat and long bones as well as the digits. (Figs. 1, 2, and 3). Otherwise normal abdomen findings.

The patient was lost to follow up after imaging diagnosis.

## Discussion

Teratoma is a congenital neoplasm derived from one or more germ cell layers: endoderm, mesoderm, and ectoderm [1], [2]–3]. Failed migration of primordial germ cells may lead to the development of teratomas [1]. There are 3 major groups of teratoma; classic type, fetiform teratoma, and malignant teratoma [2]. Mature teratomas are benign neoplasms with cystic nature, containing skin, hair follicles, teeth, and sebaceous debris [3]. Mature teratomas are commonly seen in ovaries, testes, and mediastinum, but less frequently in midline regions across the body [3]. Fetiform teratoma also known as homunculus is a rare type of mature teratomas, constituting highly organized tissues [2,3] resembling a malformed fetus but lacking the axial skeleton [1,3]. Fetiform teratoma can develop anywhere in the body but commonly in the midline, affecting the female population of 9 to 65 years of age [1]. Fetiform teratoma is an exceedingly rare entity, that about 25 cases are reported in the literature as of 2006 [1,4]. There is a variable degree of limb formation in fetiform teratoma, but lacking visceral organs or skeletal muscles [1, 4].

Another rare entity that has to be distinguished from fetiform teratoma, is a fetus in fetu. The fetus in fetu is a vertebrate fetus with an axial skeleton in a normally growing fetus [1], [2], [3], [4]–5]. Fetus in fetu almost always grows in the retroperitoneal region and presents in the early childhood period as abdominal mass [1,5] more seen in males [1]. Furthermore, cytogenetics can be used to differentiate fetus in fetu from fetiform teratoma [3,4]. Various imaging modalities are used in the evaluation of fetiform teratoma. CT scan is considered the modality of choice and a better tool for preoperative surgical planning [2]. There is a prognostic difference of fetus in fetu and fetiform teratoma, as the first is considered a benign entity while the latter has a 10% chance of malignant degeneration [1]. Complete surgical excision is the treatment of choice in fetiform teratoma [1,2]. Our case highlights the features of fetiform teratoma: highly differentiated bones resembling extremity in the lumbar region consisting of flat bones, long bones, and digits. Fetiform teratoma is a rare type of mature teratoma. It has to be distinguished from the fetus in fetu; a parasitic twin within a viable patient consisting of the axial skeleton and almost always retroperitoneal in location.

## Consent statement

Written informed consent for publication of their case was obtained from the patient

There is attached scan document of the consent along-side with this consent statement
